# A Population Growth Trend Analysis for *Neotricula aperta*, the Snail Intermediate Host of *Schistosoma mekongi*, after Construction of the Pak-Mun Dam

**DOI:** 10.1371/journal.pntd.0002539

**Published:** 2013-11-07

**Authors:** Stephen W. Attwood, E. Suchart Upatham

**Affiliations:** 1 State Key Laboratory of Biotherapy, West China Hospital, West China Medical School, Sichuan University, Chengdu, People's Republic of China; 2 Department of Life Sciences, The Natural History Museum, London, United Kingdom; 3 Faculty of Allied Health Sciences, Burapha University, Bangsaen, Chonburi, Thailand; National Institute of Parasitic Diseases China CDC, China

## Abstract

**Background:**

The Pak-Mun dam is a controversial hydro-power project on the Mun River in Northeast Thailand. The dam is sited in a habitat of the freshwater snail *Neotricula aperta*, which is the intermediate host for the parasitic blood-fluke *Schistosoma mekongi* causing Mekong schistosomiasis in humans in Cambodia and Laos. Few data are available which can be used to assess the effects of water resource development on *N. aperta*. The aim of this study was to obtain data and to analyze the possible impact of the dam on *N. aperta* population growth.

**Methodology/Principal Findings:**

Estimated population densities were recorded for an *N. aperta* population in the Mun River 27 km upstream of Pak-Mun, from 1990 to 2011. The Pak-Mul dam began to operate in 1994. Population growth was modeled using a linear mixed model expression of a modified Gompertz stochastic state-space exponential growth model. The *N. aperta* population was found to be quite stable, with the estimated growth parameter not significantly different from zero. Nevertheless, some marked changes in snail population density were observed which were coincident with changes in dam operation policy.

**Conclusions/Significance:**

The study found that there has been no marked increase in *N. aperta* population growth following operation of the Pak-Mun dam. The analysis did indicate a large and statistically significant increase in population density immediately after the dam came into operation; however, this increase was not persistent. The study has provided the first vital baseline data on *N. aperta* population behavior near to the Pak-Mun dam and suggests that the operation policy of the dam may have an impact on snail population density. Nevertheless, additional studies are required for other *N. aperta* populations in the Mun River and for an extended time series, to confirm or refine the findings of this work.

## Author Summary

There is much controversy over the effects of water resource development on the transmission of schistosomiasis in the lower Mekong Basin. Impact assessments are urgently required because there are currently 12 such projects planned in the region. The key to understanding the effects of impoundment is the impact on the snail intermediate host, which, in the case of Mekong schistosomiasis, is *Neotricula aperta*. Surprisingly, we have almost no data on *N. aperta* population trends nor on the impact of dams. To address this, the present work focused on a population near the Pak-Mun dam in Thailand. The analysis suggested that *N. aperta* populations were not growing significantly over the study period (1990–2011), but that the dam may have affected a spike in population density immediately after its completion. The study also revealed changes in density that were coincident on changes in operation of the dam; suggesting that keeping the dam open might lower snail population densities. This is the first scientific assessment of the impact of the Pak-Mun dam on *N. aperta* and suggests that dams of this kind may affect snail population density. The study also indicates an urgent need for additional independent observations and continuing regular surveys.

## Introduction

### Background

The Pak-Mun dam in Northeast Thailand, has been the focus of much controversy since the announcement of plans for its construction in 1982. Demonstrations and protests against the dam became more pronounced in 1989 when the project received government approval [1]. Construction of the dam began in June 1990 and the project was completed on 26th June 1994. Commercial operation of the 136 MW hydroelectric plant began in November 1994, but by the 9th October 1994 all four of the dam's generators were already on line and under test. The Pak-Mun project has been heavily criticized over the associated risk or flooding, displacement of villages and damage to tourist areas such as the rapids at Kaeng-Sa-Poe [1], but it is the risk of schistosomiasis transmission which is the concern of the present study.

**Figure 1:**
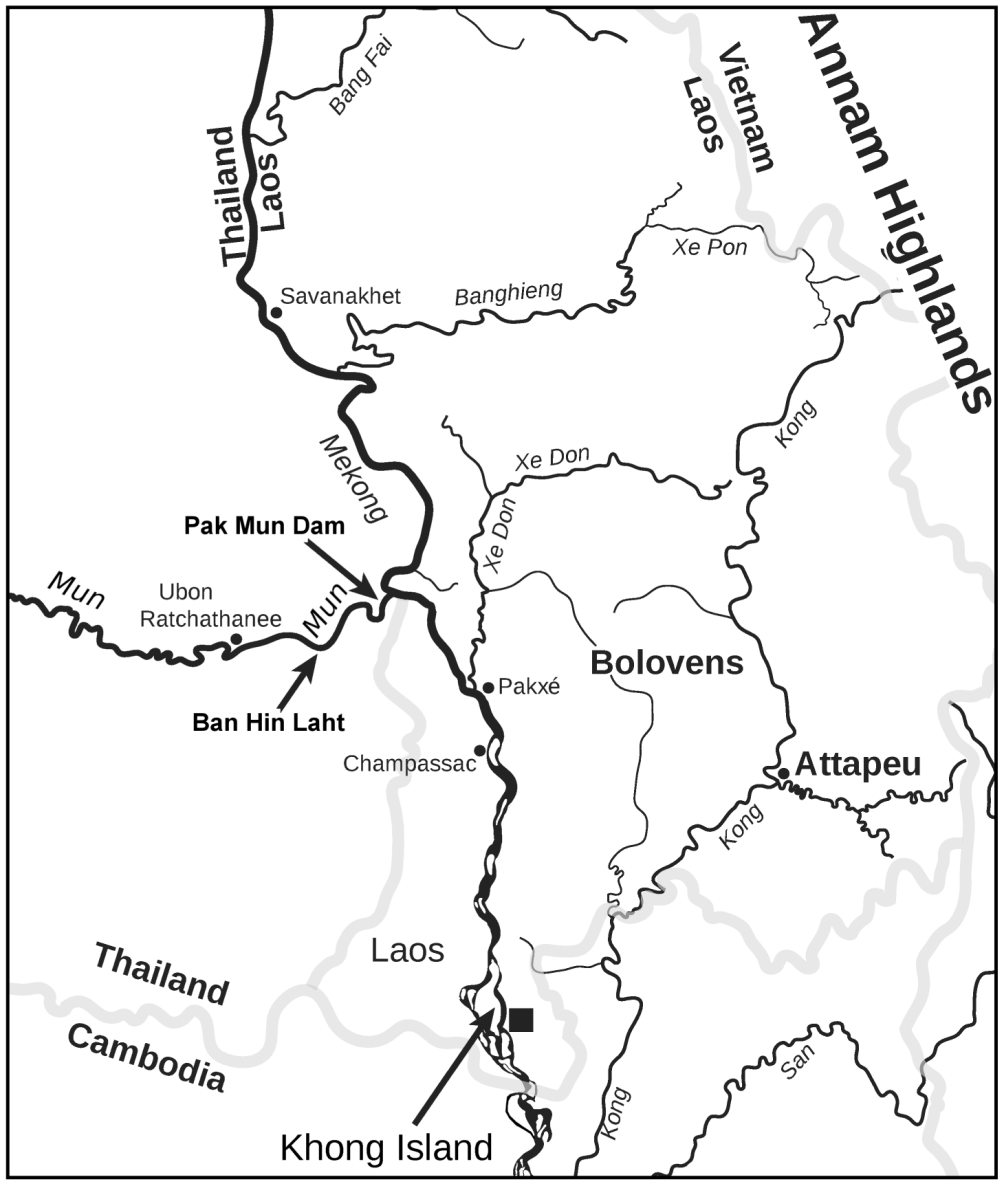
The eastern margin of the Khorat Basin showing the Mun and Mekong rivers, and other major rivers of the region. The location of the sample site (Ban Hin Laht) and the Pak-Mun dam are indicated by arrows, as is Khong Town, the closest focus of human schistosomiasis transmission to the Pak-Mun dam (the black square indicates the transmission area). Scale and international boundaries approximate.

The Pak-Mun Dam is located at Ban Hua Heo in Ubon Ratchathanee Province on the Mun river (15.281981 N 105.468095 E), 5.5 km upstream of its confluence with the Mekong river at the Lao:Thai border (Figure 1). The rocky areas around small islands in this section of the Mun river are the habitats of the epilithic rissooidean freshwater snail *Neotricula aperta* (Pomatiopsidae: Triculinae). *Neotricula aperta* is the intermediate host for the helminth blood-fluke parasite *Schistosoma mekongi* (Trematoda: Digenea) in the Mekong river and associated tributaries of Laos and Cambodia. Three strains of *N. aperta* are recognized (α, β and γ, respectively, on the basis of decreasing shell length), all three strains are able to transmit the parasite in the laboratory, but in nature only the γ-strain is found to be epidemiologically significant. The β-strain is known only from the lower Mun river and is the only strain to be found in this river (Figure S1). The average shell length of the β-strain is less than 3 mm. In laboratory studies *N. β-aperta* has been found to be the most susceptible strain to infection with *S. mekongi* [2], although this may vary depending on the source of parasite used [3]. *Schistosoma mekongi* infects humans in Laos and Cambodia and causes Mekong schistosomiasis (at foci located about 190 km from the Pak-Mun Dam, see Figure 1). Currently, *N. aperta* is known from 31 localities in Cambodia, Lao PDR and Thailand, involving nine river systems [4] and an estimated 1.5 million people are at risk of infection by *S. mekongi* [5]. Neither the disease nor infected snails have been reported from the Mun river; however, the possibility that the dam might alter ecological conditions so as to favor transmission of the parasite has been one of the main concerns regarding the Pak-Mun project [1], [6]–[9].

The major rivers of the lower Mekong Basin show a marked high water and low water seasonal flow pattern, with *N. aperta* completing its life-cycle within the low water period (March to May annually). *Neotricula aperta* is found only in shallow areas (typically 0.5 to 3 m deep) of these rivers. The snails are restricted to areas where the current is moderate (around 2×10^3^ m^3^ s^−1^), the water is clear, well oxygenated, and the bed rock forms (almost flat) platforms where algal aufwuchs is extensive [10]. Such conditions exist only during the low water period; therefore *N. aperta* persists mostly by recruitment (from eggs laid on stones in the previous year) or re-colonization from other rivers [11]. *Neotricula aperta* grazes the algal epilithon and cannot survive in areas where sediment is depositing and preventing the growth of the algae upon which it feeds. Indeed, ecological studies of *N. aperta* have shown that this snail is restricted to stones covered with fine sediments and that this species is highly sensitive to silting [12]. These “prosobranch” snails (Rissooidea) show ecological requirements that are very different from those of the pulmonate snails which transmit schistosomiasis to humans in Africa and South America; these differences have been the basis of calls for specific models to predict the effects of dam projects on the transmission of *S. mekongi*, because lessons learned from Africa and South America may not be valid in the case of Mekong schistosomiasis [13],[14].

The Pak-Mun dam is comprised of roller compacted concrete with a maximum height of 17 m and total length of 300 m. The reservoir has a surface area of 60 km^2^, at the average high water level of 108 m above the mean sea level (MSL), and a capacity of 225 million m^3^. The operating rules of the dam are designed to ensure that the water level does not exceed 106 m MSL during the low water season [1]. Consequently, the ecological conditions of the river are not much affected during the high water season but they are more greatly impacted during the low water period, which is the time when *N. aperta* is active. Following intense protest by riparian villagers in 2001, the Thai government agreed to open the gates of the Pak-Mun Dam for one year to conduct studies on the impacts of the dam on fisheries, social life and electricity supply. The study was conducted between June 2001 and July 2002 and the dam was re-closed from 1 November 2002 onwards, although the dam gates were allowed to be left open from July to October each year, thereafter, to cover the height of the flood season [15].

### Aims of the study

In spite of the many articles on line and in the popular press, which discuss the problems associated with the Pak-Mun dam project, there are very few peer-reviewed formal scientific studies available. Indeed there are only two studies on snail population density near Pak-Mun from 1996 [8], [9] and one study screening local people for Mekong schistosomiasis in 2004 [7]. The impact of the dam clearly includes attenuation of the effects of the annual flood/dry season cycle, with higher water levels, lower flow rates and (consequently) higher rates of silt deposition in the low water period. Such changes might be expected to lower *N. aperta* population densities; however, a reduction in flow rate between May and July might allow extension of the snails' breeding period and lead to an increase in population density. It is important that we understand the likely impacts of water resource development on *N. aperta* populations because plans are under consideration for at least 12 hydroelectric power dams on major rivers in the lower Mekong Basin and much controversy surrounds predictions of their environmental impacts [16]–[18].

Fortunately, one of the 1996 studies on *N. aperta* near Pak Mun [9] was performed by the present authors and used consistent snail density estimation techniques, allowing the data to be combined with those of the present study to form a time series, with frequent samples, running from 1990 to 2011. All samples were taken at a series of small islands in the Mun river near Ban Hin Laht (15.227774 N 105.271035 E) located 27 km upstream of the dam (Figure S2). The β-strain of *N. aperta* is no longer found downstream of the dam, although the γ-strain and many *Manningiella polita* snails (*M. polita* is a close relative of *N. aperta* and could be confused with *N. β-aperta*) are found at the confluence of the Mekong and Mun rivers. During the late 1990s it was not possible to take samples close to the Pak Mun dam due to political unrest. The 1996 study covered 1991 to 1995 and used ANOVA to detect any density differences among sample years, followed by pairwise comparisons between years using simple non-parametric tests. At that time no suitable method was available for analysis of short time series. Nevertheless, the study did find a statistically significant increase in densities observed after the dam came into operation compared with those seen before operation of the dam. Unfortunately, the study terminated only one year after the dam was completed and so it could give no indication of the long-term impact of the dam on snail population density.

Trends can be estimated for an observed time series using a linear mixed model expression of the Gompertz stochastic state-space exponential growth model. Modifications of this model, in the form of a linear, normal state-space model, are now available to allow for unequally spaced sampling intervals [19], [20]. The resulting model thus provides a method of trend analysis incorporating both observation error and environmental process noise, which has proven suitable for use with shorter time series and uneven sampling times (as encountered in the present investigation) [19]. Popular approaches such as ARIMA are not appropriate in the present case because these require 50 or more regular samples [21]. The approach has already proven useful in studies of *N. aperta* population changes around the Nam Theun 2 dam site in Laos [14].

In view of the unique biology and ecological niche of *N. aperta* (relative to pulmonate snails in Africa or South America), the lack of data upon which to base models, and the uncertainty as to the likely response of the snail populations to the Pak-Mun dam, further studies are clearly necessary. Consequently, the present work was undertaken in order to help fill this gap in our understanding and to investigate *N. aperta* population density changes (if any) occurring at Ban Hin Laht in the 19 years following the first operation of the Pak Mun dam. In this study snail population density estimates were collected and subjected to a trend analysis using the modified Gompertz State-Space exponential growth model. The objective was to detect any population trend (either growth or decline) and to look for any significant changes concurrent with operation of the dam.

## Methods

### Sampling

Snails were collected off stones submerged in the Mun river at Ban Hin Laht (15.227774 N 105.271035 E), the collections were a continuation of those begun in 1996 (refer to site 1, note the 1996 GPS coordinates were inaccurate) [9]. Sampling was annual or biennial for 1990–1995 and 2000–2005, with a final sample in 2011. All samples were taken within the low water period of the Mun river and within a five week period of each sampled year, before the onset of the spate which would affect the distribution of snails in the river and submerge the islands around which the populations were sampled.

The sample site was chosen because it was an area, out of the sites originally sampled in the 1996 study, of relatively low current, shallow waters and did not infringe on the fishing activities of other villagers or the boat passage (established since 1996). Consequently, the site was less risk to collector safety but covered 5480 m^2^ of river bed (measured by GPS). The area was approximately square, with five samples taken from within, one at each corner and one in the centre. The samples were taken at depths within the range 0.8 to 1.5 m. The sub-sample points were originally marked by lining up landmarks and cross referencing, but after 1999 by GPS. The beta strain of *N. aperta* is almost exclusively epilithic and is never found on or in mud or on aquatic macrophytes, consequently the snail populations could be sampled off stones collected from the river. A diver entered the water at each point and collected stones in the 113–1922 cm^2^ (surface area) range, such that approximately 0.5 m^2^ was sampled at each point. The stones were lifted out of the water directly into a plastic tray and carried back to the boat. The stones, in the trays, were left exposed to the sun for five minutes and then the stones were turned over and exposed for a further 5 minutes. The stones were then doused in water; this has the effect of causing the *N. aperta* to drop from the stones (they will otherwise try to lock the aperture of their shell to the stone and be difficult to find and dislodge). The stones were then removed, re-exposed to the sun and washed in new trays to ensure that all snails had been removed. The trays were then left in the shade for 3 minutes, during which time the snails attached themselves to the tray. The trays were then washed of any mud, sand or insects, filled with clean river water and the *N. aperta* present removed and counted. The surface area of the stones was later measured by covering the stone with plastic food wrap (cutting it to fit in a tight mono-layer); the plastic wraps were labeled and later weighed in the laboratory and a weight calibration factor used to determine the surface area. The area of any stones collected which bore no *N. aperta* was also taken into account. To further standardize the procedure all samples were taken between 10:15 am and 11:30 am. The same field personnel* were used throughout the study and the diver was restricted to only 30 seconds searching time at each point (to deter from the selection of stones with most snails attached or large stones). *In 2003 the diver was replaced by his son so as to retain the ability to sample the heavier stones.

The sampling approach involved two main assumptions; first that sampling off the stones is representative of the population density elsewhere on the river bed, and second that the site chosen is representative of all other sites along the river near to the dam. Considering the first assumption, the river bed at Ban Hin Laht is comprised of large blocks and ridges of rock incised by deep crevices. Snails are likely to cover the sides of these crevices down to the depths of sufficient light penetration, and so the true area of the sampling site is vastly greater than 5480 m^2^. In view of this, sampling off collected stones is far more representative than counting snails per unit area of sample site. It is likely that the density on the stones collected is proportional to the density on the bed rock upon which they rest because there is evidence that any stone placed in the river is rapidly colonized by *N. aperta* [22], thus snails appear to move freely between stones and other substrata. With regard to the second main assumption, the 1996 study examined inter-site variation in snail density among five sites around Ban Hin Laht (including the site of the present study) and found no significant variation (Kruskal-Wallis one-way ANOVA).

### Initial data analysis

All analyses in this study were performed using the R statistical package [23]. The sample dates were converted to weeks, with the first sample being designated as week zero. In order to determine if the counts showed a simple linear relationship with time and to assess the effects of any outliers, a simple linear regression was performed for the data. The data (snail counts per m^2^) were then screened for outliers using plots of contour lines for the Cook's distance (a measure of the influence of each data point on the overall regression result), with distances >4/n (where n is the number of observations) suggesting the presence of a possible outlier [24]. In order to check whether the data might be normally distributed a Shapiro-Wilk test was applied. Similarly, the fit of the data to a Poisson General Linear Model (GLM) and to a negative binomial GLM were also assessed.

### Parameter estimation and trend analysis

The methods used follow those of an earlier study [14], but there follows a brief outline of the approach and details of starting parameters, replicates and other conditions specific to the present study. Initially the observed *N. aperta* population densities were fitted to a modified Gompertz State-Space exponential growth model (GSS) [20], [25]–[27] and relaxed maximum likelihood estimates (REML) obtained for the four unknown parameters of the model under the standard model of deterministic exponential growth; these being:
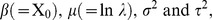
where *β* is the hypothesized (unobserved) initial value of the (log) population density (at time t = 0), *μ* is the expected change in population density over one (sampling) unit of time, *σ* ^2^ is a variance parameter representing process noise (environmental variability), and *τ* ^2^ is a variance parameter accounting for observation error. The primary aim is to estimate μ (or mu), the trend parameter, which is the rate at which median population density changes over time.

Two of the most commonly applied methods in time series analysis are the LLR model, log-linear regression of counts against time, and the Stochastic Model of Exponential Growth (SEG). The GSS is preferable to both these models. The SEG incorporates process noise and yields a log-normal probability distribution of population density, but it assumes that variability in abundances is due entirely to growth rate fluctuations caused by environmental variability (process noise). The LLR model implicitly assumes that observation error is the sole source of variation in the data (with population density governed by deterministic exponential growth). In contrast, the GSS allows for both environmental variability and sampling error. Nevertheless, the LLR and SEG models were able to provide the starting values for the four parameters of the GSS at which the REML searches were initiated. The initial value of μ (mu0) was the average of the estimate from the two models, the initial σ^2^ (ssq0) was from the SEG model, and the initial τ^2^ (tsq0) and X0 (X00) from the LLR model. The fit of the scaled GSS model was maximized using the R script for the multivariate normal log-likelihood function provided in the literature [19], which uses Nelder-Mead optimization. Ten runs of 100000 iterations each were executed with initial parameter values drawn from a normal distribution, with mean equal to the estimates from the LLR and SEG models and a SD of 1; the random number seed was also changed between each run. The REML value was noted from these runs and ten more runs performed, but scripted to only return values corresponding to a better REML. If no improvement in REML was observed, the Nelder-Mead searches were discontinued and the analysis proceeded to the McMc step described below.

GSS models can lead to a likelihood surface with multiple local maxima, such that the chance of detecting the true peak using a non-global method such as Nelder-Mead is unlikely unless the initial parameter values are very close to this peak [28]. To overcome this problem a simulated-annealing approach linked to a Metropolis sampler (SAN) was used to better explore the likelihood surface. Published studies had reported some success using the SAN approach in this context [20]. The SAN searches were run using the implementation in R (details elsewhere [14]) for 100 million generations. The model fitting was also repeated 10 times, with initial parameter values drawn from a normal distribution, with mean equal to the starting values from the LLR and SEG models and SD set such that 95% of the values drawn lay within 0.1 or 10 times the mean. The random number seed was also varied between runs. A REML frequency histogram was then plotted and the parameter estimates for models, corresponding to the minimum, mean, modal and maximum REML values, were inspected for relative variation and biological credibility. In addition, profile likelihoods were plotted (for a range of fixed mu values) to ensure that the procedure was effecting a stable and reliable estimation of the trend parameter. The parameter estimations were repeated, using the previous “best” REML estimates as starting values, until ten runs could be performed without any improvement in likelihood or change in estimated parameter values.

### Testing for zero trend

In order to determine if the snail populations showed any long-term trend (either growth or decline) a test for zero trend was performed using the standard error of the estimated mu and a standard normal percentile in an equivalence testing framework [29]. The null hypothesis being that a significant trend is present (i.e., the mu estimate lies outside a fixed, specified, interval containing zero). The equivalence region was obtained by simulating the data 1 million times, with mu set to zero and then allowing the GSS (with SAN) to provide REML estimates of mu for these simulated data sets. The simulation procedure was that used by previous authors [19]. The starting values for the other three parameters were the REML estimates (see above), with the error terms (E and F) drawn from normal distributions with mean zero and SD ssq and tsq, respectively. The exponential growth equation is then (on the log scale):
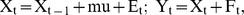
where Yt represents our observations at time t, whilst Xt represents the true population density that we cannot observe (only sample), and the simulated population densities are exp(Yt). The 95% confidence interval (CI, i.e., ±t. SE(mu)) for the 1 million mu values was then used as the equivalence region. A naïve bootstrap procedure was used to generate a 97% CI for mu (equivalent to a 95% CI in a two-tailed test). The original data were bootstrapped 1 million times using the “sample()” function in R (i.e., simple sampling with replacement), the time series was re-ordered by week, and if any two weeks (observations) had the same value, one week was added to the latter observation. REML estimates were then obtained using the GSS and SAN as described above. If the 95% CI for the empirical (bootstrapped) mu lay entirely outside that for the simulated mu values, then the null hypothesis that a substantial trend is present would be tentatively accepted. The procedure is not ideal because the same model is used to estimate mu and to simulate the data for the equivalence region, also the time series is short and the empirical cumulative distribution function (cdf) after bootstrapping may not be a good approximation to the true cdf. Nevertheless, the method avoids the subjective decisions of conventional equivalence testing, such as what the upper and lower bounds of the equivalence region should be for a snail population little studied previously in this respect.

### Predicting snail population densities

It was considered necessary to derive a work flow by which population densities could be predicted and CIs obtained for these predictions. A Kalman filter was used to yield optimized estimates of population density according to the scaled GSS model and REML parameter estimates. The filter estimates the current value of the population density at time t (Xt) under the scaled GSS, given the history of the Yt values up to and including Yt [20]. The R script used to implement the filter was taken from the literature [19]; however, this procedure assumes that the population under study is far from equilibrium (e.g., it is relatively recently established). The history of the *N. aperta* populations in the Mun river is not known and the populations could have been fluctuating around the habitat's carrying capacity for some time. Consequently, the Kalman filter was modified to assume stationarity [20] and predictions for both stationary and non-stationary cases were made (here stationary refers to the joint distribution of all Yt). The CI for these predictions were obtained by bootstrapping, following the exact same procedure as for the CI for mu (described above).

## Results

### Fitting conventional linear models

The data consisted of 9 time series observations for the period from 1990 to 2011, during which the snail population density ranged from 300 to 2108 m^−2^ (Table 1). The data were first subjected to a simple linear regression (SLR). The SLR gave no indication that the slope of the regression was significantly different from zero (*P* = 0.7959); however, the F ratio (0.0722) was not significant (Table 2), suggesting that the SLR might not be a suitable model for these data. In addition, plots of residual errors against their fitted values were not random, QQ-plots were not normal and leverage plots showed large Cook's distances for the 1991 and 1995 observations (see Figure S3). The critical Cook's distance for these data was 0.444; this was exceeded by the 1991 observation (distance almost 1.0) and approached by the 1995 observation (distance ∼0.40). Consequently, the 1991 observation was classed as an outlier, with the 1995 datum retained as a borderline observation. The 1991 observation was unusually low (300 m^−2^) and the 1995 observation unusually high (2108 m^−2^), with the mean population density being 1177 m^−2^. Table 1 shows the effect of removal of the outlier on the mean and standard deviation (SD). A Poisson general linear model (GLM) was also fitted to the data. The result was highly significant (Table 2), indicating that the GLM was, like the SLR, not a good model for describing these data. The quotient of residual variance over degrees of freedom for the Poisson GLM was 237 and 69.65 (for the full data set and without outlier, respectively), suggesting that the counts were over dispersed. In view of this the negative bionomial GLM, another Poisson-based model, was applied and found to be a better fit to the data than either SLR or Poisson GLM (Table 2). Unlike the Poisson GLM, the residual deviance for the negative binomial was not significant (*P* = 0.232 and 0.237, full data set and without outlier respectively). The negative binomial for the data without the outlier indicated a significant slope to the trend (−0.0004606); however, the slope for the full data set was not significantly different from zero (*P* = 0.771).

**Table 1:** Summary statistics for the time series observations of *Neotricula aperta* population density in the Mun river at Ban Hin Laht.

Data Set	No. of observations in time series	Range (m^−2^)	Mean population density m^−2^ ± SD
Full	9	300–2108	1177.11±476.04
Excluding outlier	8	979–2108	1286.75±367.89

The sampling period was 1990–2011.

**Table 2:** Fit of conventional linear models to the population density estimates for *Neotricula aperta*.

	Simple Linear Regression			GLM (Poisson/Negative Binomial)			
Data	Equation (Density = )	Student's-*t*	*P*	Null deviance	Resid. deviance	AIC	*P*
Full	−0.1277t+1235.702	−0.269	0.796	1676/9.370	1661/9.292	1744/142.67	*/.
Excluding outlier	−0.5756t+1580.048	−1.726	0.135	663.1/13.66	417.9/8.015	493.6/115.99	*/.

For the Simple Linear Regression, t represents time in weeks from the first sample (t = 0). *P* values:. >0.2,

* <0.0001.

The equation for the negative binomial is exp(−0.0001189t+7.1245608), full data, and exp(−0.0004606t+7.382287) excluding outlier.

In view of the fact that the 1991 observation was clearly an outlier and that there was no *a priori* hypothesis to predict the low population density in that year, the analyses proceeded with both the full data set and one with the 1991 observation omitted. The SLR for the partial data set was also not significant (but showed a slightly better fit to the data partial data set than to the full set) and, like the full data set, a negative slope was indicated (Table 2). The GLM was also rejected for the partial data set. The Shapiro-Wilke Normality Test for the full data set suggested that the data might be normally distributed (W = 0.9162, *P* = 0.3615); however, that for the partial data set indicated that the data were not normally distributed (W = 0.7909, *P* = 0.02286).

### Trend parameter estimation

Independent runs of REML searches (coupled to SAN) were found to converge on the same parameter estimates and REML values, regardless of the starting parameters (which were either the output of a run of ML searches (each based on the output of the previous search) or random variants around the LLR and SEG estimates). Also, improvement in restricted likelihood was found after 10 runs of 1 million iterations each (with different starting parameter values). Consequently, it is likely that the parameter estimates reported (Table 3) are the best estimates or close to them. Plots of log likelihood against mu also suggested that maximum likelihood estimates had been found (Figure S4).

**Table 3:** REML parameter estimates for the “best-fit” GSS model found by Nelder-Mead/SAN.

Data set	mu	ssq	tsq	exp(X0)	ln(REML)
Full	1.52350e-04	0.00000	0.31661	994.0777	−6.46414
Excl outlier	−3.92380e-04	3.23506e-03	2.39343	1521.465	−16.83423

As seen in Table 3, after fitting the modified Gompertz linear state space model (GSS), the REML estimate of the trend parameter (mu) for the full data set was positive, whilst that for the partial data set was negative, although the 95% confidence intervals (CI) for the two estimates overlapped one another (Table 4); the mu estimate in both cases was very small. The REML for the full data set fitted to the GSS was −6.464139 and that for the partial data set was −16.83423.

**Table 4:** Confidence intervals (95%) for estimates of mu, with the “best-fit” GSS model, based on the original (empirical) data set, from bootstrapped data sets and from simulations (where mu = 0).

Data set	mu (empirical)	C.I. for mu (empirical)	C.I. for simulations	C.I. bootstrapped data
Full	1.52350e-04	−8.8316e-04 to 1.1879e-03	−5.8264e-04 to 5.8440e-04	−9.9404e-04 to 1.3043e-03
Excl outlier	−3.92380e-04	−5.0638e-03 to 4.2791e-03	−5.2471e-03 to 5.2493e-03	−8.5623e-04 to 1.3125e-04

### Hypothesis testing

In the case of the full data set, the empirical (bootstrapped) CI completely overlapped the equivalence region, but exceeded it at both ends. The empirical mu (1.52350e-04) lay well inside the upper limit of the equivalence region (CI for the simulations with mu fixed at zero (see Figure S5)). From this it is reasonable to conclude that the true mu might be >0 but is certainly close to or equal to zero. In contrast, with the partial data set, the CI for the empirical mu lay entirely within the equivalence region; therefore the hypothesis that mu is significantly different from zero may be rejected at *P*<0.05.

### Predictions

Using the REML parameter estimates, and under the GSS model, the Kalman filter was employed to predict the true population density (X_t_) for both the stationary and non-stationary cases (Table 5). The predictions and the observed (original) data (Y_t_) are plotted in Figure 2. The Kalman filter was also used to predict values for key years and CIs for these predictions obtained by bootstrapping the data 1 million times. For these predictions only the partial data set was used because the 1991 observation was unlikely to have been a reflection of natural events or consistent with long-term environmental processes. Similar predictions were also made using the SLR and GLM models, where statistically appropriate. The predicted values from the negative binomial GLM were very close to those of the Poisson GLM and so only the values for the negative binomial are given in Table 5 (as the negative binomial was the statistically preferred model of the two). The similar predictions from the two GLM models is not unexpected because the negative binomial mainly differs from the Poisson GLM in the way in which the likelihood function penalizes class values with high variances, and this difference may not always be sufficient to be reflected in parameter estimates; however, it is likely to affect their variance [30].

**Figure:**
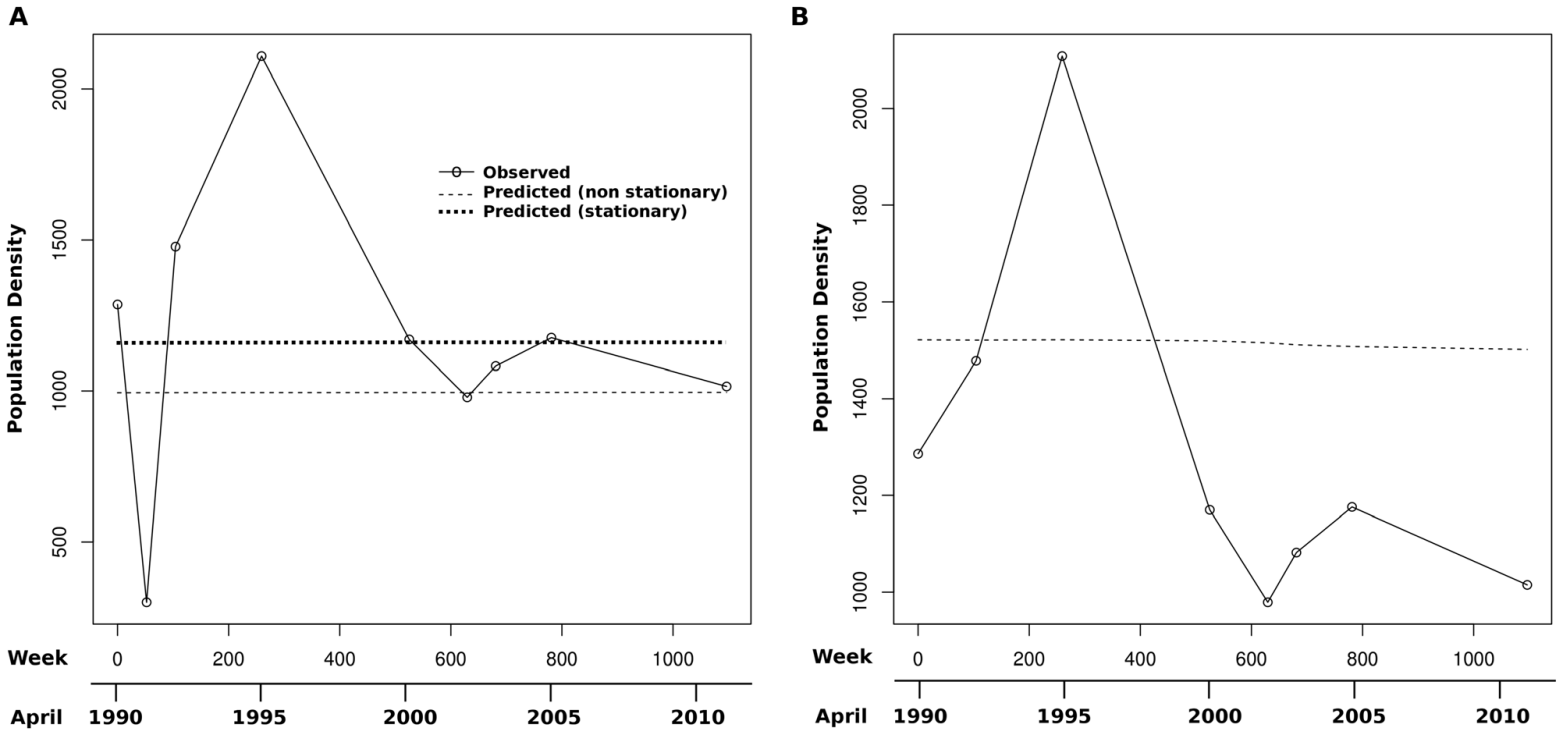
Plots of population density against sampling time for A, the full data set and B, the data with the outlying (1991) observation removed. The original observations are plotted, as well as GSS model predicted values for both the non-stationary and stationary cases. The stationary case plot is omitted from B because it is identical to that for the non-stationary plot at this scale (see Table 5). Time was measured in weeks, but an additional scale in years is given for convenience.

**Table 5:** Model-based predictions of the 1991, 1995 and 2002 population densities.

Year	SLR	GLM (Negative Binomial)	GSS (non-stationary)	GSS (stationary)
1991	1549.944±922.0065	1569.009±171.8124	1408.522±493.2719	1408.522±493.2719
1995	1270.881±329.0185	1265.686±49.1193	1270.391±302.1927	1270.362±302.2005
2002	1121.328±319.2501	1202.776±84.12235	1295.712±588.5236	1295.707±588.5239
2020	807.571±451.6727	781.541±166.5251	1216.884±244.0057	1216.884±244.0061

Values are given ± a 95% confidence interval (two-tailed); those for SLR and GLM are based on the standard error of the regression and those of the GSS on bootstrap resampling. The observed densities were 300 m^−2^, 2108 m^−2^, and 979 m^−2^ for 1991, 1995 and 2002 respectively. Predictions were made using the data set excluding the 1991 observation. The time series was also extrapolated to give the expected density in 2020 under each model.

The trend was extrapolated and the snail population density in 2020 predicted. This prediction is based on observed densities up to and including the 2011 observation, and assumes the same growth trend operates from 2012 to 2020 as operated between 1990 and 2011. The population density at Ban Hin Laht in 2020 was predicted to be 1216.884±244.0057 which is very close to, and not significantly different from, the average population density observed today (*P*<0.05). It should be noted, however, that the predicted values are estimates of the true population density (X_t_) rather than the expected observed density (Y_t_). In contrast, the SLR and GLM models predicted that population densities would be much lower in 2020 than at present. The 1991 population density was also predicted using the GSS and estimated at 1408.522±493.2719 (Table 5); this is significantly greater than the observed density of 300 and confirms that the 1991 observation was atypical and must reflect some event occurring at Ban Hin Laht prior to sampling. Predictions were also made for 1995 and 2002, years for which data were available; however, these are key years because 1995 is the first year after dam operation and the 2002 sample was taken after the dam had been kept open for almost one year. In these cases initiating the Kalman filter under non-stationary or stationary assumptions had a small effect on the predictions. The 1995 observation (2108 m^−2^) was significantly greater than the predicted value (1270.391±302.1927) and this was also found with the SLR and GLM models. In contrast, the 2002 observation was less than the predicted density (979 m^−2^ *cp.* 1295.712±588.5236 m^−2^); however, this was not statistically significant with any model, except for the negative binomial GLM which predicted a value significantly lower than the observed density (Table 5).

## Discussion

No significant large population growth trend was indicated at Ban Hin Laht, either with the full or partial data set. Under the GSS model the partial data set indicated that the population growth parameter was not significantly different from zero. Consequently, the data support the view that population densities have remained fairly constant over the sampling period. Furthermore, predictions of the 2020 population density, based on parameters estimated using the GSS model, were that the density in 2020 will not be significantly different from the average density observed between 1990 and 2011.

The observed population density for 1991 was less than a quarter of the value predicted using any model (SLR, GLM or GSS). This observation was taken in the first dry season after construction of the Pak-Mun dam began (see Table 6). It is possible that silt, debris or toxic materials could have been washed from the construction site and affected the viability of *N. aperta* eggs deposited at Ban Hin Laht. The study site is only 33 km from the confluence with the Mekong river and flow reversal could occur at Ban Hin Laht at the height of the flood period in the Mekong river. Indeed, Mekong river backflow 70–100 km upstream from the confluence has been predicted for the Nam Songkhram river [31]. Further, *N. aperta* is known to be sensitive to depositing silt and prefers a fine eroding substratum [12]. An alternative explanation is that there was some undocumented attempt to control the snails (probably using molluscicides) after local villagers began to protest over the risk of schistosomiasis. By the following April the *N. aperta* population density recovered to levels slightly exceeding the study average, suggesting that, if the dam construction had effected the drop in density, the impact was associated with the early stages of construction (most likely the initial movements of earth and excavations).

**Table 6:** Time line of dam construction and operational changes for the Pak-Mun dam 1994–2011.

Year	Status	Flow rate affected
1990 (June)	Project construction begins	No
1993	Project structures in place, however dam was not licenced to impound	No
1994	Installation of generators and testing	Yes (intermittently)
1994 (Nov)	Dam begins to produce electricity	-
1995–2001	Dam operating year round (with gates closed to some degree)	Yes
2001	All dam gates kept open June 2001–July 2002	No*
2002	All dam gates remained open during *N. aperta* breeding season	No
2003-present	Dam operating, but all gates kept open July to October	Yes

The right-most column indicates whether the river flow rate upstream of the dam (near the sampling area) is likely to have been affected during the active phase of the *Neotricula aperta* life-cycle of that year (i.e., the low-water period March to May); the low-water target elevation of the upstream waters at the dam site was 105.5 m (MSL). Sources [1], [15].

* The dam being open in June would have prevented the more rapid and unnatural termination of the low-water period (and activity of *N.* aperta), which would have occurred under the usual dam operation policy.

The 1995 observation was taken in the second dry season following completion of the dam (Table 6), and is the time of the highest population density observed in this study. The observed density was almost double (and statistically significantly greater than) the predicted values with either the SLR, GLM or GSS models, and so indicates a marked departure from the overall population trend running through the study period. Between 1992 and1995 the Pak-Mun dam effected a shift toward slightly more lentic conditions at the study site (a reduction in flow rate and increase in depth [9]). It is conceivable that reduced flow rates and delaying of the onset of the annual flood could increase survival and breeding success in *N. aperta* and this may have led to the increase in population density observed; however, by 2002 densities had fallen to levels below average and below GSS and SLR predictions (although not significantly). The high densities in 1995 could be explained by a process similar to that observed shortly after filling of artificial water impoundments, where fish stocks of many indigenous species rise in the short-term before declining in the longer term; this is due to factors such as expansion into new ecological niches (that are not the usual niche of the species concerned) in the absence of competitors and barriers such as high current [32]. The 1995 observation might also be explained as increased sampling efficiency due to ease of diving in slower flowing waters than those usually encountered when making observations at Ban Hin Laht; however, this implies that densities recorded during 2002–2011 should also be high, unless some overriding factor were acting to reduce snail populations during those years.

The fluctuations in observed density might be explained by sampling bias due to population growth or decline across the sampling period (which ranged from April 7 to May 10). During this time interval, which lies well within the low water (dry season) period, snail populations in the Mun River have not been observed to increase in density and are quite stable, but the snails do increase in shell size. This could slightly increase sampling efficiency in May relative to April (because larger snails might be easier to remove from the stones); however, initial investigations into the design of the sampling procedure suggested that even juvenile snails were removed by the technique used in this study. In contrast the increase in shell size by May could effect a lower on-stone snail density because fewer snails could be accommodated on the same sized stone, although at times of very high observed densities the snails were found to climb on top of one another and graze the shells of other snails in the same manner as they would graze the stone itself – even though suitable areas of the stone's surface were still not yet fully occupied. In any case, both high and low densities were recorded in April and May of different years, so that there did not appear to be any trend correlated with sampling month. For example, the highest and the lowest observed densities both occurred in early April.

The 2002 observation was much lower than the density predicted by any model; however, this was not statistically significant with any model except the GLM. This fall in density was observed after the dam operation had been changed to keep the gates open (allowing near normal flow of the Mun river) for the preceding two years; thus suggesting that keeping the dam open might lower snail population densities. It is tempting to attribute this fall to the year round opening of the dam, especially as the population density returns to that of normal dam operation levels after the policy change to gate closure during the flood period. Opening of the dam could limit snail population growth by allowing the natural onset of the spate, at least one month earlier than when the gates are closed, which terminates the breeding period of *N. aperta*. The fall in density was, however, not statistically significant and there are many other factors which could explain the decline (for example, natural cycles in *N. aperta* population density or that of competitor species, local pollution from sources unrelated to Pak-Mun and increases in Mekong river flood volume).

In conclusion, this study has shown that there has been no marked increase in *N. aperta* population growth following operation of the Pak-Mun dam and long-term projections are that over the ten years following the study snail population densities will remain close to their current average. Indeed, the growth trend parameter estimated for the time series observations was not statistically different from zero, implying that *N. aperta* populations near Pak-Mun were quite stable. Nevertheless, the analysis does indicate that changes in snail population density did co-occur with changes in operation of the dam, although only two out of three of these changes were statistically significant (i.e., 1991, 1995). The present study is, however, limited in its scope and the applicability of its findings. The study examined population changes at only one site on the Mun river and changes observed might be peculiar to Ban Hin Laht. Similarly, no samples were taken downstream of Pak-Mun where the ecological impacts of the dam are expected to be quite different. Confidence in the conclusions of studies of this kind will be greatly improved by further work at additional sites around the dam (if data are available) and by extending the present data set with further, regular, observations beyond 2011. In view of the fact that many dams are now planned across the range of *N. aperta* and *S. mekongi*, it is vital that we better understand the effects of dams such as Pak-Mun on snail population growth. The present work suggests that studies of this kind are useful in understanding the impacts of water impoundment but data are currently severely lacking. Investment in research into this area is urgently required to enable the planning of public health compliant water resource development in the lower Mekong Basin.

## Supporting Information

Checklist S1 STROBE Checklist.
(DOC)

Figure S1 *Neotricula β-aperta* on a stone collected from the Mun river near Ban Hin Laht.
(JPG)

Figure S2 The study site at Ban Hin Laht on 11 May 2002.
(JPG)

Figure S3 Plots of residual errors against their fitted values, scale-location, QQ-plots and leverage plots (showing Cook's distances) from a simple linear regression of the full data set.
(PDF)

Figure S4 Plot of likelihood against fixed mu value, for a range of mu values about the REML estimate of mu obtained by Nelder-Mead optimization (full data set). The range is the 95% confidence interval for the REML estimate of mu.
(PNG)

Figure S5 Frequency plot for simulated mu (when true mu is zero).
(PNG)
