# Supplementary material for: A Population Growth Trend Analysis for Neotricula aperta, the Snail Intermediate Host of Schistosoma mekongi, after Construction of the Pak-Mun Dam
Source: PLoS Negl Trop Dis. 2013 Nov 7;7(11):e2539. doi: 10.1371/journal.pntd.0002539 (PMC3820754; doi:10.1371/journal.pntd.0002539)
Supplement: Checklist S1 — STROBE Checklist. (DOC) [file pntd.0002539.s001.doc]

STROBE Statement—Checklist of items that should be included in reports of ***cohort studies***

Authors' comment: *The study was an ecological study rather than an epidemiological study (or a cohort study); however, in recognition of the fact that many of the Strobe recommendations are relevant to all observational studies, we are submitting this check-list.*

|  | Item No | Recommendation |
| --- | --- | --- |
| **Title and abstract** | 1 | (*a*) Indicate the study’s design with a commonly used term in the title or the abstract  Yes |
|  |  | (*b*) Provide in the abstract an informative and balanced summary of what was done and what was found Yes |
| Introduction | | |
| Background/rationale | 2 | Explain the scientific background and rationale for the investigation being reported Yes |
| Objectives | 3 | State specific objectives, including any prespecified hypotheses Yes |
| Methods | | |
| Study design | 4 | Present key elements of study design early in the paper Yes (Methods: Sampling sites, reasons for site choice, sampling procedure are described) |
| Setting | 5 | Describe the setting, locations, and relevant dates, including periods of recruitment, exposure, follow-up, and data collection Yes (sample sites described, also sampling dates given and those of changes in dam operation policy) |
| Participants | 6 | (*a*) Give the eligibility criteria, and the sources and methods of selection of participants. Describe methods of follow-up N/A |
|  |  | (*b*) For matched studies, give matching criteria and number of exposed and unexposed N/A |
| Variables | 7 | Clearly define all outcomes, exposures, predictors, potential confounders, and effect modifiers. Give diagnostic criteria, if applicable N/A |
| Data sources/ measurement | 8* | For each variable of interest, give sources of data and details of methods of assessment (measurement). One variable – snail population density, source of data was direct counting of snails within a known area. Describe comparability of assessment methods if there is more than one group N/A. |
| Bias | 9 | Describe any efforts to address potential sources of bias: Fixed sampling time duration and time of day, fixed season of sampling |
| Study size | 10 | Explain how the study size was arrived at (Study size: sampled the available, relevant, known, accessible population; sample size was the maximum possible under restrictions – sampling disturbed the distribution of individuals and so must be completed in one day |
| Quantitative variables | 11 | Explain how quantitative variables were handled in the analyses. If applicable, describe which groupings were chosen and why: Modified Gompertz State Space Model with accommodation of terms for observation error and environmental fluctuations |
| Statistical methods | 12 | (*a*) Describe all statistical methods, including those used to control for confounding  Modified Gompertz State Space Model, General Linear Models |
|  |  | (*b*) Describe any methods used to examine subgroups and interactions N/A |
|  |  | (*c*) Explain how missing data were addressed: Used a Gompertz State Space Model modified to accommodate missing data |
|  |  | (*d*) If applicable, explain how loss to follow-up was addressed N/A |
|  |  | (*e*) Describe any sensitivity analyses N/A |
| Results | | |
| Participants | 13* | (a) Report numbers of individuals at each stage of study—eg numbers potentially eligible, examined for eligibility, confirmed eligible, included in the study, completing follow-up, and analysed N/A |
|  |  | (b) Give reasons for non-participation at each stage N/A |
|  |  | (c) Consider use of a flow diagram N/A |
| Descriptive data | 14* | (a) Give characteristics of study participants (eg demographic, clinical, social) and information on exposures and potential confounders: They were all beta strain *Neotricula aperta* snails |
|  |  | (b) Indicate number of participants with missing data for each variable of interest N/A |
|  |  | (c) Summarise follow-up time (eg, average and total amount) N/A |
| Outcome data | 15* | Report numbers of outcome events or summary measures over time: Yes (Snail population density reported for each sample year) |
| Main results | 16 | (*a*) Give unadjusted estimates and, if applicable, confounder-adjusted estimates and their precision (eg, 95% confidence interval). Make clear which confounders were adjusted for and why they were included: Yes (Snail population densities were estimated by GLM and GSS models to give values adjusted for sampling error and environmental fluctuations) |
|  |  | (*b*) Report category boundaries when continuous variables were categorized N/A |
|  |  | (*c*) If relevant, consider translating estimates of relative risk into absolute risk for a meaningful time period N/A |
| Other analyses | 17 | Report other analyses done—eg analyses of subgroups and interactions, and sensitivity analyses N/A |
| Discussion | | |
| Key results | 18 | Summarise key results with reference to study objectives Yes |
| Limitations | 19 | Discuss limitations of the study, taking into account sources of potential bias or imprecision. Discuss both direction and magnitude of any potential bias Yes |
| Interpretation | 20 | Give a cautious overall interpretation of results considering objectives, limitations, multiplicity of analyses, results from similar studies, and other relevant evidence Yes |
| Generalisability | 21 | Discuss the generalisability (external validity) of the study results: Full details of the analytical procedures are given so that re-analyses are possible. Raw data are given in Figure 2. |
| Other information | | |
| Funding | 22 | Give the source of funding and the role of the funders for the present study and, if applicable, for the original study on which the present article is based Yes |

*Give information separately for exposed and unexposed groups.

**Note:** An Explanation and Elaboration article discusses each checklist item and gives methodological background and published examples of transparent reporting. The STROBE checklist is best used in conjunction with this article (freely available on the Web sites of PLoS Medicine at http://www.plosmedicine.org/, Annals of Internal Medicine at http://www.annals.org/, and Epidemiology at http://www.epidem.com/). Information on the STROBE Initiative is available at http://www.strobe-statement.org.
