# Supplementary figures and images for: A Population Growth Trend Analysis for Neotricula aperta, the Snail Intermediate Host of Schistosoma mekongi, after Construction of the Pak-Mun Dam
Source: PLoS Negl Trop Dis. 2013 Nov 7;7(11):e2539. doi: 10.1371/journal.pntd.0002539 (PMC3820754; doi:10.1371/journal.pntd.0002539)

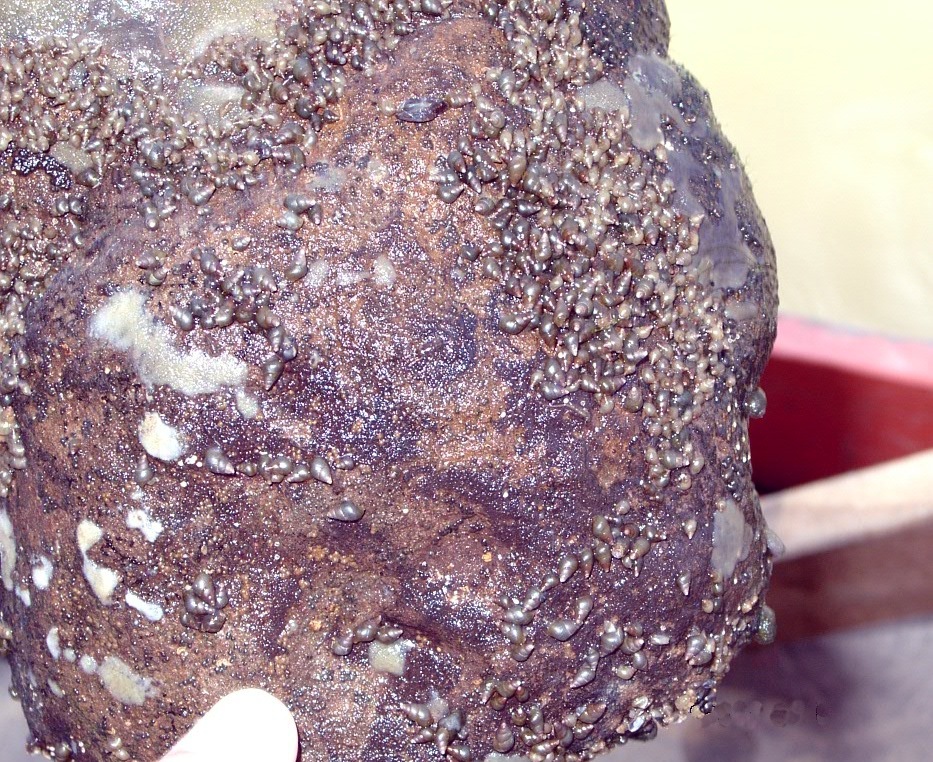

Supplement: Figure S1 — Neotricula β-aperta on a stone collected from the Mun river near Ban Hin Laht. (JPG) [file pntd.0002539.s002.jpg]

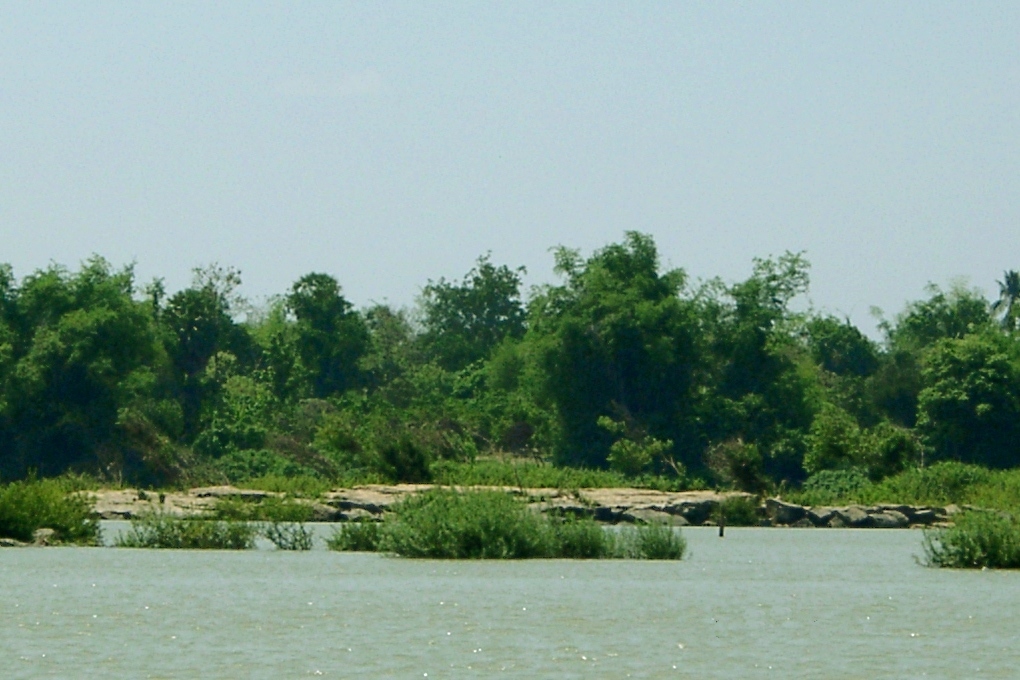

Supplement: Figure S2 — The study site at Ban Hin Laht on 11 May 2002. (JPG) [file pntd.0002539.s003.jpg]

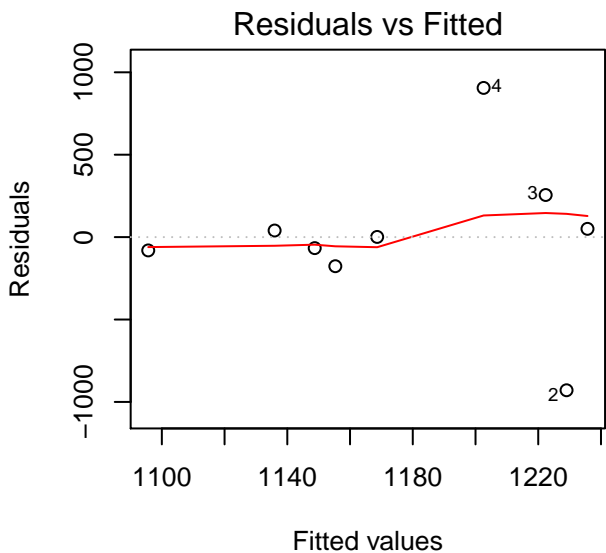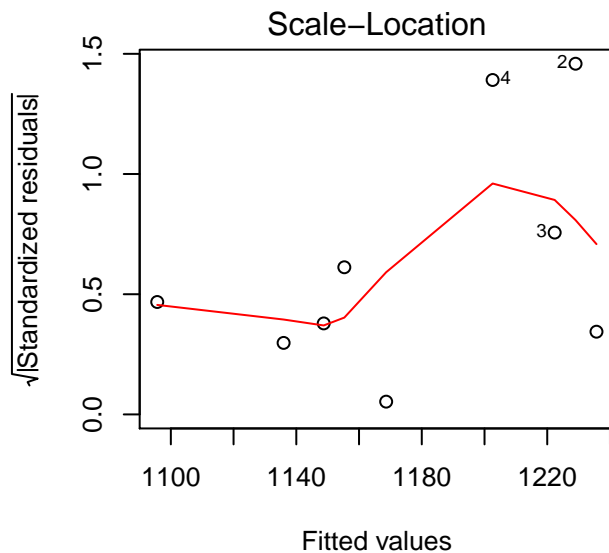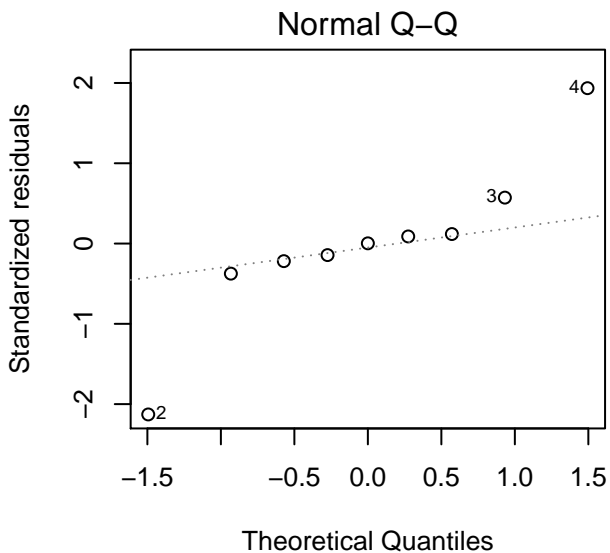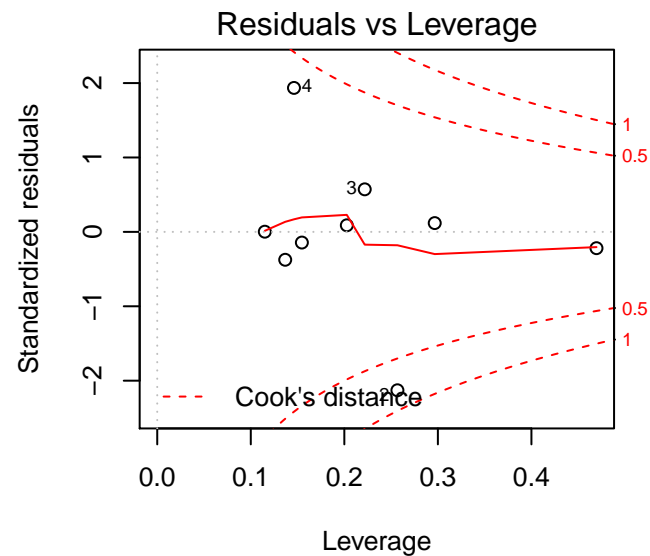

Supplement: Figure S3 — Plots of residual errors against their fitted values, scale-location, QQ-plots and leverage plots (showing Cook's distances) from a simple linear regression of the full data set. (PDF) [file pntd.0002539.s004.pdf]

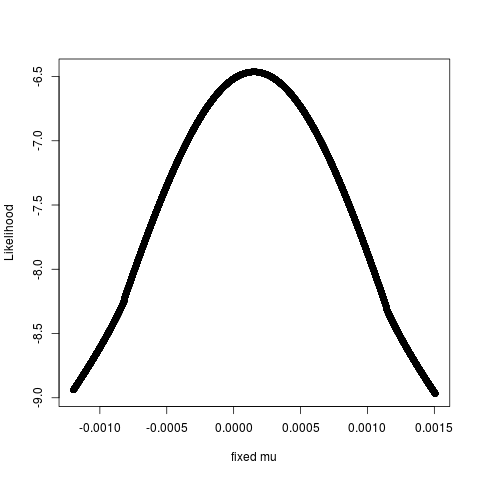

Supplement: Figure S4 — Plot of likelihood against fixed mu value, for a range of mu values about the REML estimate of mu obtained by Nelder-Mead optimization (full data set). The range is the 95% confidence interval for the REML estimate of mu. (PNG) [file pntd.0002539.s005.png]

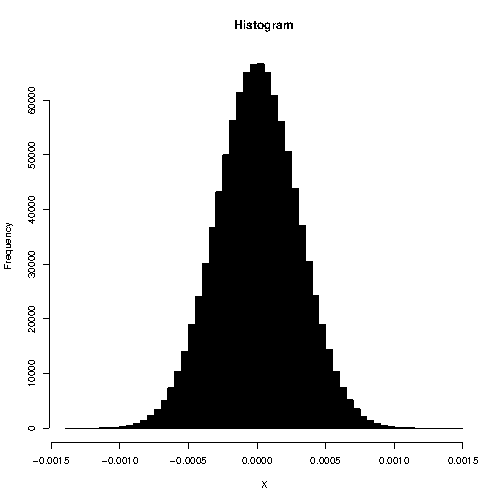

Supplement: Figure S5 — Frequency plot for simulated mu (when true mu is zero). (PNG) [file pntd.0002539.s006.png]
